# Supplementary material for: Angle resolved photoemission spectroscopy reveals spin charge separation in metallic MoSe2 grain boundary
Source: Nat Commun. 2017 Feb 6;8:14231. doi: 10.1038/ncomms14231 (PMC5303875; doi:10.1038/ncomms14231)
Supplement: Supplementary Information — Supplementary Figures, Supplementary Notes and Supplementary Equations [file ncomms14231-s1.pdf]

Supplementary Note 1: Characterisation of charge density wave in mirror twin grain boundaries

Charge density waves (CDW) are instabilities of the Fermi surface in the presence of electron-phonon coupling. The ideal case is when all electrons can be excited, which implies that all parts of Fermi surfaces are connected by the same q-vector. This property of a Fermi surface is called perfect nesting. For an ideal 1D system, the Fermi surface consists of two points (or two parallel lines), at $-k_F$ and $+k_F$ (where k_F is the Fermi wave vector), and thus exhibits perfect nesting at $q = 2k_F$. Hence, all one-electron (1D) systems are subject to CDW transitions for which this phenomenon is also known as Peierls transition. The periodicity of the charge density is given by π/k_F. The Peierls transition is also often described by a change of the Brillouin zone (BZ) due to the change of the lattice periodicity to π/k_F i.e. the BZ boundary now coincides with k_F. Therefore a band gap opens and the Peierls transition is associated with a metal-to-insulator transition. Fig. 2 (c) shows the characteristics of the CDW/Peierls transition.

In the scanning tunnelling microscopy (STM) measurements the grain boundaries appear as two parallel lines[1], and can be assigned to the position of the Se−atoms neighbouring the defect line. The STM contrast is consistent with calculations[2] that show charge density expanding into vacuum at the Se atom positions adjacent to the defect. At room temperature, the measured corrugation along these Se-rows corresponds to the atomic lattice (a_{MoSe_2} = 3.3 Å). Imaging the MTBs at lower temperatures causes their periodicity to increase to $3\times a_{MoSe_2}$, as shown in Figs. 2 (a) and (b). Such an increase in periodicity in STM is consistent with the formation of a charge density wave (CDW)[3]. Thus the STM measurement suggests $k_F = \frac{\pi}{3}\frac{1}{a_{MoSe_2}} \approx 0.32$ Å^{-1} , which is in good agreement with Fermi-surface mapping by ARPES. The high resolution momentum resolved photoemission data show k_F/π= 0.30 Å^{-1}.

The metal - insulator transition (MIT) associated with the CDW causes an increase of the electrical resistance of the sample. To probe this and to determine the transition temperature we performed simple four-point resistance measurements. For four point measurements a slightly thicker $MoSe_2$ film was grown of 1-2 monolayer thick to ensure connectivity between the $MoSe_2$ islands. Contacts to the sample were made by silver paint with contacts separated by ≈ 1mm. Standard van der Pauw geometry for four point measurements was adopted and the temperature dependent sample resistance was measured in a cryogenic probe station cooled with liquid nitrogen. During the measurement the sample current was kept constant and the potential drop was evaluated to determine the sample resistance. This procedure implies that as the sample resistance decreases at lower T the applied potential increases to maintain a constant current. For a critical applied potential a de-pinning of the CDW is then observed.

Consistent with MIT(s) our 4-point measurements, shown in Fig 2 (d), exhibit a resistance jump at ≈ 235 K and a second smaller increase at ≈ 200 K. The occurrence of multiple transitions is attributed to incommensurate and commensurate CDW transitions[4]. In addition, we also observed a drop of the resistance for a critical applied voltage (see Fig. 2 (d)). This behaviour is known from CDW in quasi-1D bulk crystals[5,6] and is caused by a de-pinning of the charge density wave from defects and a collective sliding of CDW. While CDW transition are expected for 1D electron systems (1DES), it is no proof for it.

Supplementary Note 2: Broadening of the ARPES spectral function and analysis of the EDC, MDC and lifetime

It is well-know that in 1D metallic systems there are no quasi-particles in the vicinity of the Fermi surface. The excitations rather are gapless collective modes involving charge and spin degrees of freedom. All these features have dramatic consequences, particularly on the angle-resolved photoelectron spectroscopy (ARPES) directly measured spectral function of interacting 1D fermions. Specifically, any structure of the spectral function other than the Fermi-energy cut-off, is noticeably broad. This is another typical hallmark of spin-charge separation due to the fact that there are no stable excitations with the quantum numbers of the electron. The broadening of the peaks occurs both at low energy near the Fermi level and at higher energies. Effectively, this broadening of the photoemission peaks is a hallmark of the spin-charge separation.

All theses concepts have been robustly probed in the early ARPES time, see for more details Ref. [7]. Due to the separation of charge and spin, one hole (or one electron) is always unstable to decay into two or more elementary excitations, of which one or more carries its spin and one or more carries its charge. Consequently, the ARPES spectral function does not have a pole contribution, but rather consists of a multi-particle continuum. As it has been detailed in the method section of this article, the exploration of the shape of energy distribution curves (EDC) and momentum distribution curves (MDC) plots shows clearly the enlargement of the lifetime, which can be originated by a spin-charge separation. This approximative evaluation is just proportional to various interaction strengths present in the system.

Supplementary Note 3: Definition and validity of some quantities used in the theoretical analysis

On the one hand, the distributions $2t\,\eta_{\rm c}(\Lambda)$ and $2t\,\eta_{\rm s}(\Lambda)$ appearing in the expressions of the pseudofermion energy dispersions $\varepsilon_{\rm c}(q)$ and $\varepsilon_{\rm s}(q')$ given in Eq. (8) are the unique solutions of the coupled integral equations provided in the supplementary Equations (1) and (2) where $u = U/4t > 0$.

On the other hand, the q and q' dependence of the dispersions $\varepsilon_{\rm c}(q)$ and $\varepsilon_{\rm s}(q')$ occurs through that of the momentum rapidity function $k = k(q)$ for $q \in [-\pi, \pi]$ and spin rapidity function $\Lambda = \Lambda(q')$ for $q' \in [-k_{\rm F}, k_{\rm F}]$, respectively. Those are defined in terms of their inverse functions $q = q(k)$ for $k \in [-\pi, \pi]$ and $q' = q'(\Lambda)$ for $\Lambda \in [-\infty, \infty]$ given in the supplementary Equations (3) and (4), respectively. The distributions $2\pi\rho(k)$ and $2\pi\sigma(\Lambda)$ in these equations are the unique solutions of the coupled integral equations provided in the supplementary Equations (5) and (6).

Within the $\beta = c, s$ pseudofermion representation, the conformal-field theory dressed-charge matrix and the corresponding matrix $(Z^{-1})^T$ can be expressed in terms of pseudofermion phase shifts in units of 2π, as given in the supplementary Equation (7). For the zero-spin density case considered in the article, such matrices can be written as in the supplementary Equation (8).

In the zero spin-density case, the spin $SU(2)$ symmetry implies that the parameter $\xi_{\rm s}$ appearing in supplementary Equation (8) is u independent and given by $\xi_{\rm s} = \sqrt{2}$. The parameter $\xi_{\rm c}$ in that equation in turn reads $\xi_{\rm c} = f(\sin Q/u)$ where the function $f(r)$ is the unique solution of the integral equation given in the supplementary Equation (9). In it, Q is the parameter defined in the supplementary Equation (3) and the function $D(r)$ is provided in the supplementary Equation (10) where $\Gamma(x)$ is the usual Γ function.

The rapidity phase shifts $2\pi\bar{\Phi}_{\rm c,c}(r, r')$ and $2\pi\bar{\Phi}_{\rm c,s}(r, r')$ in the expressions of the c pseudofermion phase shifts $2\pi\Phi_{\rm c,c}\,(q, q') = 2\pi\bar{\Phi}_{\rm c,c}\,(\sin k(q)/u, \sin k(q')/u)$ and $2\pi\Phi_{\rm c,s}\,(q, q') = 2\pi\bar{\Phi}_{\rm c,s}\,(\sin k(q)/u, \Lambda(q')/u)$ considered in the Methods section are the unique solutions of the integral equations given in the supplementary Equations (11) and (12) where $D(r)$ and $D_0(r)$ are defined in supplementary Equations (10) and (13), respectively.

The related s pseudofermion phase shifts $2\pi\Phi_{\rm s,c}(\iota k_{\rm F}, q)$ and $2\pi\Phi_{\rm s,s}(\iota k_{\rm F}, q')$ are provided in the supplementary Equations (14) and (15), respectively. (The corresponding more general s pseudofermion phase shifts $2\pi\Phi_{\rm s,s}(q', q)$ and $2\pi\Phi_{\rm s,c}(q', q)$ are uniquely defined by the solution of integral equations that are not given here.)

Concerning the validity of the spectral functions expressions in Eqs. (9) and (18), when for an electron removal spectral function γ branch line there is for $(\omega_\gamma(k) - \omega) < 0$ (ii) no spectral weight and (ii) a very small amount of weight, they are (i) exact and (ii) a very good approximation for an energy window corresponding to a small energy deviation $(\omega_\gamma(k) - \omega) > 0$ from the high-energy branch-line spectrum $\omega_\gamma(k)$. In the present case, the electron removal spectral function expressions are exact for the s branch line and a very good approximation for the k ranges of the c and c$'$ branch lines for which the corresponding power-law exponents are negative, respectively.

Supplementary Note 4: The electron finite-range interactions versus onsite interactions only

Can the approach used in the paper to account for the effects of the electron finite-range interactions be used to extract information beyond that given by the conventional 1D Hubbard model about the physics of quasi-1D metals? For each finite effective U/t and electronic density $n < 1$, the value of the c$'$ branch-line exponent $\tilde{\zeta}_{\rm c'}(k)$ in Eq. (19) decreases upon increasing α within the range $\alpha \in [\alpha_0, 1/8]$ and increases upon further increasing it within the interval $\alpha \in]1/8, \alpha_{\rm max}]$. Consistently, there is a α value belonging to the interval $\alpha \in]1/8, \alpha_{\rm max}]$ for which the c$'$ branch line momentum width where the exponent $\tilde{\zeta}_{\rm c'}(k)$ is negative exact equals that obtained for the conventional 1D Hubbard model at $\alpha = \alpha_0$. Within the renormalized pseudofermion dynamical theory introduced in the paper, this may be behind the agreement reported in Refs. [8,9] of the spectral features predicted by that model pseudofermion dynamical theory with those observed in the quasi-1D metal tetrathiafulvalene tetracyanoquinodimethane (TTF-TCNQ) corresponding to the the TCNQ molecular chains[10,11]. A RPDT description would imply a $K_{\rm c} < 1/2$ TLL charge parameter for such chains. By using the conventional 1D Hubbard model, the studies of Refs. [8,9] rather assumed that $K_{\rm c} > 1/2$ for the TCNQ molecular chains.

For the 1D Hubbard model with electron finite-range interactions corresponding to $\alpha = 0.78$, the theoretical c branch line spectrum $\omega_{\rm c}(k) = \varepsilon_{\rm c}(|k| + k_{\rm F})$, c$'$ branch line spectrum $\omega_{\rm c'}(k) = \varepsilon_{\rm c}(|k| - k_{\rm F})$, and s branch line spectrum $\omega_{\rm s}(k) = \varepsilon_{\rm s}(k)$ are plotted in Fig. 5 (d) for $U/t = 0.8$, $t = 0.58\,{\rm eV}$ and $n = 2/3$. The corresponding branch-line spectra of the conventional 1D Hubbard model are plotted for these values in Supplementary Figure 5. The c, c$'$, and s branch lines appear in the latter figure as full and dashed lines for k ranges for which the corresponding exponents are negative and positive, respectively.

The difference between Fig. 5 (d) and Supplementary Figure 5 refers mainly to the momentum range for which the c$'$ branch line exponent is negative, which is inexistent for the 1D Hubbard model with finite-range interactions corresponding to $\alpha = 0.78$. An additional difference is that for the latter model the s branch line exponent becomes

positive for k values near the $\pm k_{\rm F}$ Fermi points.

Supplementary Equations

$$2t\,\eta_{\rm c}(k) = 2t\sin k + \frac{\cos k}{\pi\,u}\int_{-\infty}^{\infty}d\Lambda\,\frac{2t\,\eta_{\rm s}(\Lambda)}{1+\left(\frac{\sin k-\Lambda}{u}\right)^2}\,. \qquad \text{Supplementary Equation (1)}$$

$$2t\,\eta_{\rm s}(\Lambda) = \frac{1}{\pi\,u}\int_{-Q}^{Q}dk\,\frac{2t\,\eta_{\rm c}(k)}{1+\left(\frac{\Lambda-\sin k}{u}\right)^2} - \frac{1}{2\pi\,u}\int_{-\infty}^{\infty}d\Lambda'\,\frac{2t\,\eta_{\rm s}(\Lambda')}{1+\left(\frac{\Lambda-\Lambda'}{2u})\right)^2}\,. \qquad \text{Supplementary Equation (2)}$$

$$\begin{aligned} q(k) &= k + \frac{1}{\pi}\int_{-\infty}^{\infty}d\Lambda\,2\pi\sigma(\Lambda)\,\arctan\left(\frac{\sin k-\Lambda}{u}\right) \quad \text{for} \quad k\in[-\pi,\pi]\,, \\ & q(\pm Q) = \pm 2k_{\rm F}\,; \qquad \pm Q = k(\pm 2k_{\rm F})\,, \\ & q(\pm\pi) = \pm\pi\,; \qquad \pm\pi = k(\pm\pi)\,. \end{aligned} \qquad \text{Supplementary Equation (3)}$$

$$\begin{aligned} q'(\Lambda) &= \frac{1}{\pi}\int_{-Q}^{Q}dk\,2\pi\rho(k)\,\arctan\left(\frac{\Lambda-\sin k}{u}\right) \\ &- \frac{1}{\pi}\int_{-\infty}^{\infty}d\Lambda'\,2\pi\sigma(\Lambda')\,\arctan\left(\frac{\Lambda-\Lambda'}{2u}\right) \quad \text{for} \quad \Lambda\in[-\infty,\infty]\,, \\ & q'(\pm\infty) = \pm k_{\rm F}\,, \qquad \pm\infty = \Lambda(\pm k_{\rm F})\,. \end{aligned} \qquad \text{Supplementary Equation (4)}$$

$$2\pi\rho(k) = 1 + \frac{\cos k}{\pi\,u}\int_{-\infty}^{\infty}d\Lambda\,\frac{2\pi\sigma(\Lambda)}{1+\left(\frac{\sin k-\Lambda}{u}\right)^2}\,. \qquad \text{Supplementary Equation (5)}$$

$$2\pi\sigma(\Lambda) = \frac{1}{\pi\,u}\int_{-Q}^{Q}dk\,\frac{2\pi\rho(k)}{1+\left(\frac{\Lambda-\sin k}{u}\right)^2} - \frac{1}{2\pi\,u}\int_{-\infty}^{\infty}d\Lambda'\,\frac{2\pi\sigma(\Lambda')}{1+\left(\frac{\Lambda-\Lambda'}{2u})\right)^2}\,. \qquad \text{Supplementary Equation (6)}$$

$$\begin{aligned} Z &= \begin{bmatrix} 1 & 0 \\ 0 & 1 \end{bmatrix} + \sum_{\iota=\pm1}\begin{bmatrix} \Phi_{\rm c,c}(\iota 2k_{\rm F},2k_{\rm F}) & \Phi_{\rm c,s}(\iota 2k_{\rm F},k_{\rm F}) \\ \Phi_{s,c}(\iota k_{\rm F},2k_{\rm F}) & \Phi_{s,s}(\iota k_{\rm F},k_{\rm F}) \end{bmatrix}\,, \\ (Z^{-1})^T &= \begin{bmatrix} 1 & 0 \\ 0 & 1 \end{bmatrix} + \sum_{\iota=\pm1}(\iota)\begin{bmatrix} \Phi_{\rm c,c}(\iota 2k_{\rm F},2k_{\rm F}) & \Phi_{\rm c,s}(\iota 2k_{\rm F},k_{\rm F}) \\ \Phi_{s,c}(\iota k_{\rm F},2k_{\rm F}) & \Phi_{s,s}(\iota k_{\rm F},k_{\rm F}) \end{bmatrix}\,. \end{aligned} \qquad \text{Supplementary Equation (7)}$$

$$Z = \begin{bmatrix} \xi_{\rm c} & \xi_{\rm c}/2 \\ 0 & 1/\xi_{\rm s} \end{bmatrix}\,; \quad (Z^{-1})^T = \begin{bmatrix} 1/\xi_{\rm c} & 0 \\ -\xi_{\rm s}/2 & \xi_{\rm s} \end{bmatrix}\,; \quad \xi_\beta = \sqrt{2K_\beta} \quad \text{for} \quad \beta = c,s\,. \qquad \text{Supplementary Equation (8)}$$

$$f(r) = 1 + \int_{\frac{-sinQ}{u}}^{\frac{sinQ}{u}}dr'D(r-r')\,f(r')\,. \qquad \text{Supplementary Equation (9)}$$

$$D(r) = \frac{1}{\pi}\int_0^{\infty}d\omega\frac{\cos(\omega\,r)}{1+e^{2\omega}} = \frac{i}{2\pi}\frac{d}{dr}\ln\frac{\Gamma\left(\frac{1}{2}+i\frac{r}{4}\right)\Gamma\left(1-i\frac{r}{4}\right)}{\Gamma\left(\frac{1}{2}-i\frac{r}{4}\right)\Gamma\left(1+i\frac{r}{4}\right)}\,. \qquad \text{Supplementary Equation (10)}$$

$$2\pi\bar{\Phi}_{\rm c,c}(r,r') = -D_0(r-r') + \int_{-\frac{\sin Q}{u}}^{\frac{\sin Q}{u}}dr''\,D(r-r'')\,2\pi\bar{\Phi}_{\rm c,c}(r'',r')\,. \qquad \text{Supplementary Equation (11)}$$

$$2\pi\bar{\Phi}_{c,s}(r,r') = -\arctan\Big(\sinh\Big(\frac{\pi}{2}(r-r')\Big)\Big) + \int_{-\frac{\sin Q}{u}}^{\frac{\sin Q}{u}} dr''\, D(r-r'')\, 2\pi\bar{\Phi}_{c,s}(r'',r')\,. \qquad \text{Supplementary Equation (12)}$$

$$D_0(r) = 2\int_0^\infty d\omega \frac{\sin(\omega\, r)}{\omega(1+e^{2\omega})} = i\,\ln\frac{\Gamma\Big(\frac{1}{2}+i\frac{r}{4}\Big)\Gamma\Big(1-i\frac{r}{4}\Big)}{\Gamma\Big(\frac{1}{2}-i\frac{r}{4}\Big)\Gamma\Big(1+i\frac{r}{4}\Big)}\,. \qquad \text{Supplementary Equation (13)}$$

$$2\pi\Phi_{s,c}(\iota k_F, q) = -\frac{\iota\,\pi}{\sqrt{2}} \text{ for } \iota = \pm 1\,. \qquad \text{Supplementary Equation (14)}$$

$$2\pi\Phi_{s,s}(\iota k_F, q') = \iota\,\pi\frac{(\xi_s-1)(\xi_s+(-1)^{\delta_{q',\iota k_F}})}{\xi_s} = \frac{\iota\,\pi}{\sqrt{2}}(\sqrt{2}-1)(\sqrt{2}+(-1)^{\delta_{q',\iota k_F}}) \text{ for } \iota = \pm 1\,. \qquad \text{Supplementary Equation (15)}$$

---

[1] Liu, H., Jiao, L., Yang, F., Cai, Y., Wu, X., Ho, W., Gao, C., Jia, J., Wang, N., Fan, H., Yao, W., Xie, M. Dense Network of One-Dimensional Midgap Metallic Modes in Monolayer $MoSe_2$ and Their Spatial Undulations. *Phys. Rev. Lett.* **113**, 066105 (2014).

[2] Lehtinen, O., Komsa, H.-P., Pulkin, A., Whitwick, M.B., Chen, M.-W., Lehnert, T., Mohn, M.J., Yazyev, O.V., Kis, A., Kaiser, U., Krasheninnikov, A.V. Atomic Scale Microstructure and Properties of Se-Deficient Two-Dimensional $MoSe_2$. *ACS Nano* **9**, 3274-3283 (2015).

[3] Barja, S., Wickenburg, S., Liu, Z.-F., Zhang, Y., Ryu, H., Ugeda, M.M., Hussain, Z., Shen, Z.-X., Mo, S.-K., Wong, E., Salmeron, M.B., Wang, F., Crommie, M.F., Ogletree, D.F., Neaton, J.B., Weber-Bargioni, A. Charge density wave order in 1D mirror twin boundaries of single-layer $MoSe_2$. *Nature Phys.* DOI: 10.1038/NPHYS3730 (2016).

[4] Sinchenko, A. A., Monceau P. Dynamical transport properties of NbSe3 with simultaneous sliding of both charge-density waves. *Phys. Rev. B* **87**, 045105 (2013).

[5] Grüner, G. The dynamics of charge density waves. *Rev. Mod. Phys.* **60**, 1129-1181 (1988).

[6] Thorne, R.E. Charge-density-wave conductors. *Phys. Today* **49**, 42-47 (1996).

[7] Valla, T., Fedorov, A. V., Johnson, P. D., Hulbert S. L. Many-body effects in angle-resolved photoemission: quasiparticle energy and lifetime of a Mo(110) surface state *Phys. Rev. Lett.* **83**, 2085-2088 (1999).

[8] Carmelo, J. M. P., Penc, K., Sacramento, P. D., Sing, M., Claessen, R. The Hubbard model description of the TCNQ related singular features in photoemission of TTF-TCNQ. *J. Phys.: Condens. Matter* **18** 51915212 (2006).

[9] Carmelo, J. M. P., Bozi, D., Penc, K. Dynamical functions of a 1D correlated quantum liquid. *J. Phys.: Condens. Matter* **20**, 415103 (2008).

[10] Claessen, R., Sing, M., Schwingenschlgel, U., Blaha, P., Dressel, M., Jacobsen, C.S. Spectroscopic Signature of Spin-Charge Separation in the quasi-one-dimensional organic conductor TTF-TCNQ. *Phys. Rev. Lett.* **88**, 096402 (2002).

[11] Sing, M., Schwingenschlögl, U., Claessen, R., Blaha, P., Carmelo, J. M. P., Martelo, L. M., Sacramento, P. D., Dressel, M., Jacobsen, C. S. Electronic structure of the quasi-one-dimensional organic conductor TTF-TCNQ. *Phys. Rev. B* **68**, 125111 (2003).

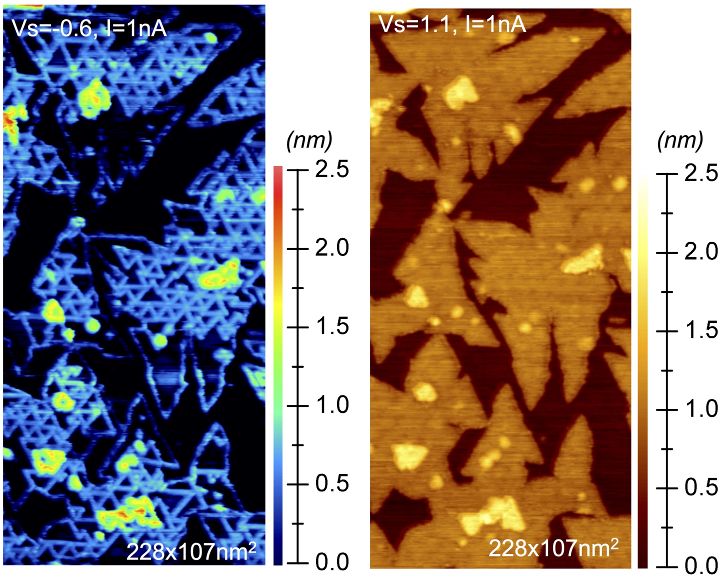

Supplementary Fig. 1. **Scanning tunnelling micrographs of submonolayer $MoSe_2$.** The imaged islands are predominantly monolayer thick $MoSe_2$ flakes, which have been used in ARPES measurements. The STM data are recorded of the same surface area with different tunnelling conditions are shown. One image emphasizes the metallic MTBS and island edges, the other tunnelling conditions emphasizes the island shape and topography. tunnelling conditions are indicated in the figures.

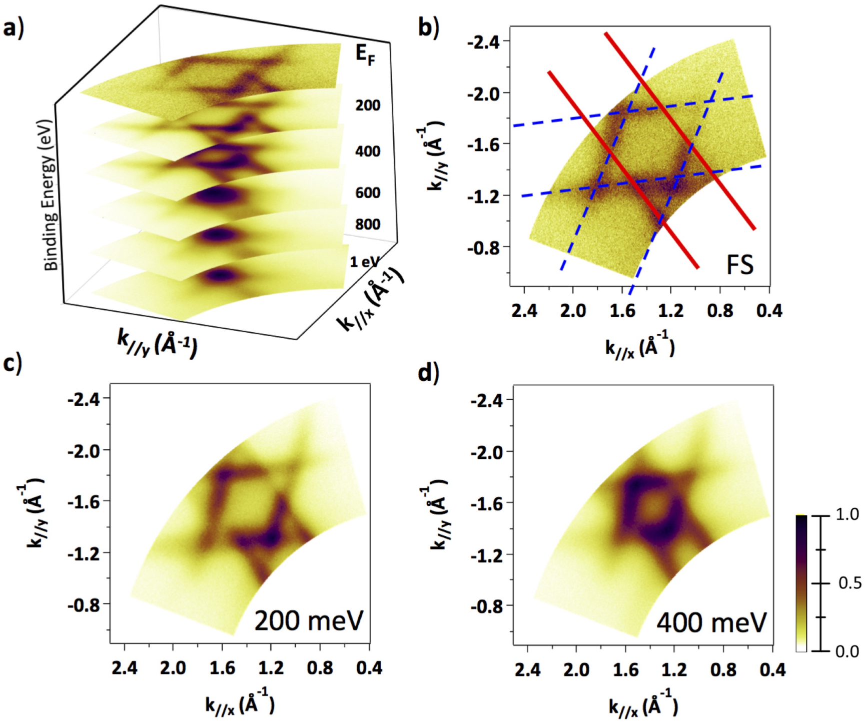

Supplementary Fig. 2. **Fermi-surface and other constant energy surfaces of MTBs.** Experimental measurements of a constant energy surfaces at different binding energies are represented in a 3D plot in (a). Spectral function around the Γ-point at the Fermi level and binding energies of 200 meV and 400 meV are plotted in (b) (c) and (d), respectively. The data shown were obtained with circular polarized light and the intensity for left and right polarized light are added together. The measurements are close to the 4-point of the 2nd BZ of the $MoSe_2$ unit cell.

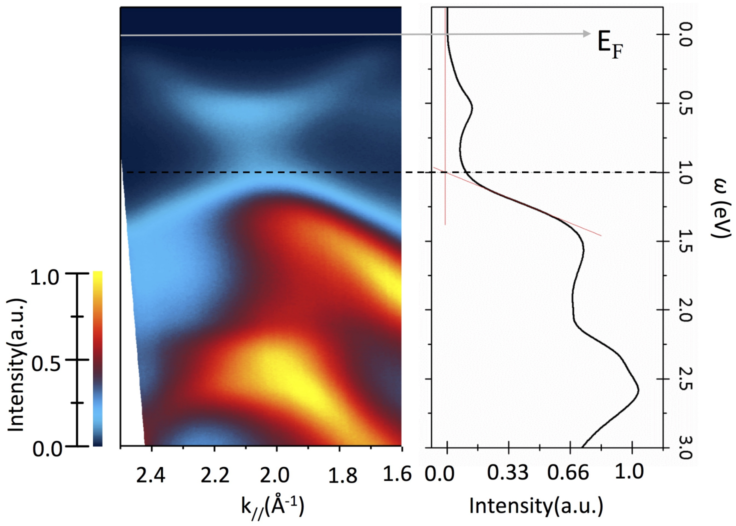

Supplementary Fig. 3. **Gap and top of the valence band of $MoSe_2$ flakes with line defects** Wide energy range photoemission spectrum around Gamma point showing the 1DES and the valence band of the $MoSe_2$ host material. The 1DES lies entirely within the band gap of the semiconducting $MoSe_2$, thus no overlap of electronic states are observed that could affect the interpretation of the 1DES.

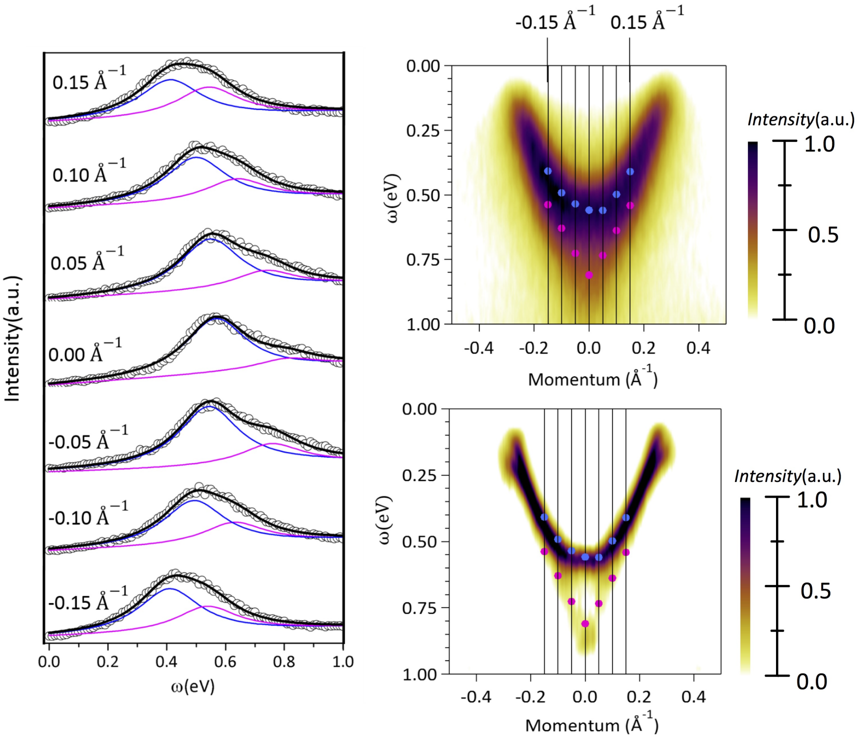

Supplementary Fig. 4. **Selected energy distribution curves (EDCs).** The EDCs contain spectral weight that cannot be fit with a single component. A fit using two components with a Lorentzian line shape and by assuming a linear background is shown on the left. The fitted peak positions are shown on the right superimposed on the spectral intensity map as well as on the processed data that shows the cusps in the second derivative of the data. The peak positions determined from fitting with two peaks agree well with the band dispersion obtained by calculating the second derivatives of the data.

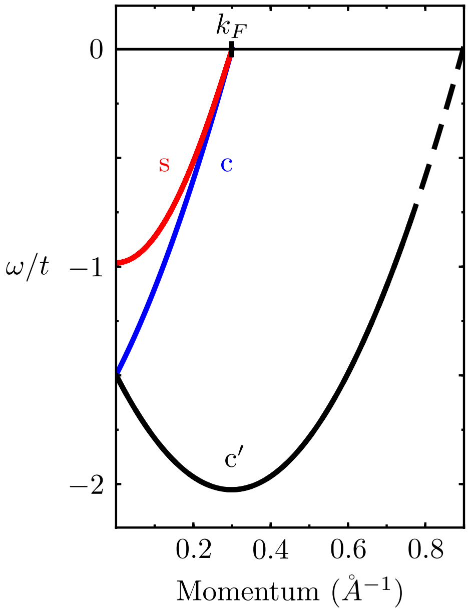

Supplementary Fig. 5. **Conventional Hubbard model branch-lines spectra.** The same c, c′ and s branch line spectra as in Fig. 5 (d) now for the conventional 1D Hubbard model with the same $U/t = 0.8$, $t = 0.58\,\text{eV}$ and $n = 2/3$ values. The full and dashed lines refer to momentum ranges for which the corresponding exponents are negative and positive, respectively. The differences relative to Fig. 5 (d) lay in the momentum range for which the c′ branch line exponent is negative, which is absent in the case of the model renormalised by finite-range interactions corresponding to $\alpha = 0.78$, and the s branch line exponent of the conventional 1D Hubbard model being negative for its whole momentum range.
